# Polycyclic Aromatic Hydrocarbons Adsorption onto Graphene: A DFT and AIMD Study

**DOI:** 10.3390/ma11050726

**Published:** 2018-05-03

**Authors:** Bing Li, Pengfei Ou, Yulan Wei, Xu Zhang, Jun Song

**Affiliations:** 1 School of Engineering, Huzhou University, Huzhou 313000, China; weiyl@zjhu.edu.cn; 2 Department of Mining and Materials Engineering, McGill University, Montreal, QC H3A 0C5, Canada; 3 School of Qiuzhen, Huzhou University, Huzhou 313000, China; 02201@zjhu.edu.cn

**Keywords:** graphene, polycyclic aromatic hydrocarbons, adsorption, hydrophobic, strain engineering

## Abstract

Density functional theory (DFT) calculations and ab-initio molecular dynamics (AIMD) simulations were performed to understand graphene and its interaction with polycyclic aromatic hydrocarbons (PAHs) molecules. The adsorption energy was predicted to increase with the number of aromatic rings in the adsorbates, and linearly correlate with the hydrophobicity of PAHs. Additionally, the analysis of the electronic properties showed that PAHs behave as mild n-dopants and introduce electrons into graphene; but do not remarkably modify the band gap of graphene, indicating that the interaction between PAHs and graphene is physisorption. We have also discovered highly sensitive strain dependence on the adsorption strength of PAHs onto graphene surface. The AIMD simulation indicated that a sensitive and fast adsorption process of PAHs can be achieved by choosing graphene as the adsorbent. These findings are anticipated to shed light on the future development of graphene-based materials with potential applications in the capture and removal of persistent aromatic pollutants.

## 1. Introduction

Polycyclic aromatic hydrocarbons (PAHs) are series of organic compounds containing only carbon and hydrogen elements which are composed of two or more fused aromatic rings [1]. PAHs originate from a variety of sources, such as incomplete combustion of coal, petroleum products, wood and organic polymer compounds [2,3]. Due to their chemical stability and low degradability, PAHs may accumulate in the soil. However, the residual PAHs in soil are of great health concern as human exposure to environment with low-level PAHs may cause increased risk of cancer, mutagenesis and teratogenicity [4,5]. Therefore, it is of urgency and importance to develop efficient processing technologies to remove PAHs in the soil [6,7,8]. With the advantages of low cost, simple operation and high efficiency, adsorption has been recognized as a practical approach to treat organic pollutants in the soil [9]. Adsorption of PAHs on carbonaceous adsorbents, in particular carbon nanomaterials and their modified forms, has been actively investigated in the past decades [10,11,12,13]. The carbon nanomaterials, including fullerenes, single- and multi-walled carbon nanotubes (CNTs), and graphene (Gr), have been demonstrated to have great potential in adsorbing organic pollutants [14,15,16,17].

Since the successful exfoliation of Gr in 2004 [18], it has been reported recently to show exquisite performance in capture of many organic pollutants, such as PAHs and their derivatives [16,19,20,21,22,23,24,25,26], antibiotics [27], dyes [28,29], and pesticides [30]. Specifically, the adsorption characteristics of various PAHs onto Gr and its oxide surfaces have been widely studied under various conditions of pH, temperature or humic acid (HA) [16,19,20,21,22,23]. For instance, Zhao et al. [24] and Shen et al. [25] have demonstrated sulfonated graphene as a superb adsorbent that possesses high adsorption capacities and fast adsorption rates for a few PAHs (e.g., naphthalene, phenanthrene and methylene blue) in aqueous solutions. In addition, Wang et al. [26] investigated the potential adsorptive sties and molecular mechanisms of naphthalene, phenanthrene, and pyrene onto Gr and its oxide, and confirmed that the interactions with PAHs might change the conformations and influence the adsorption sites. Despite those tremendous experimental efforts, there have been limited computational and theoretical studies to focus on understanding the mechanism underlying the adsorption of PAHs onto Gr. Kozlov et al. [31] demonstrated that PAHs can be adsorbed onto Gr with large adsorption strength and high stability. In addition, particular PAHs can also induce a bandgap opening of Gr which is sufficient to modify Gr as semiconductors at room temperature [32,33].

There has been no theoretical study so far to report the relationship between the adsorption strength and hydrophobicity of PAHs when adsorbed onto Gr and the simulation of dynamic adsorption process under the ambient condition. Therefore, in this study, the adsorption mechanisms between 16 PAHs and Gr were studied by density functional theory (DFT) calculations together with ab-initio molecular dynamics (AIMD) simulations. The overall objectives are: (1) to determine the most stable configurations of PAHs adsorbed onto Gr; (2) to elucidate the effects of PAHs adsorption on the electronic properties of Gr; (3) to discuss the strain engineering on the adsorption strengths between PAHs and Gr; and (4) to simulate the dynamic adsorption process by AIMD simulations. This study highlights the adsorption characteristics between PAHs and Gr and is expected to pave the way for applications in resolving environmental concerns.

## 2. Computational Methods and Details

### 2.1. Density Functional Theory (DFT) Calculations

The adsorption characteristics of PAHs molecules onto Gr were systematically studied by first-principles DFT calculations using the Vienna Ab-initio Simulation Package (VASP) [34,35,36]. The generalized gradient approximation (GGA) parametrized by Perdew–Burke–Ernzerhof (PBE) functional was chosen to describe the exchange–correlation interactions [37]. The DFT calculations with dispersion correction is known as a reasonable and low-cost choice to estimate the weak interactions as compared to other ultrahigh-accuracy computational methodologies (such as MP2 method [38]). Therefore, the PBE functional with the DFT-D correction method was employed in this study (zero damping DFT-D3 method of Grimme [39,40,41]) to characterize the weak van der Waals (vdW) interactions in the adsorption systems (local density approximation (LDA) as a comparison [42]). An 8 × 4 orthogonal Gr unit was established as the adsorbent, and the periodic boundary conditions were used in the adsorption systems. 15 Å vacuum layer was added in the vertical direction to avoid the interactions between two neighboring Gr images. The dielectric constant of the simulation box was set to the value of water (78.54) to account for the aqueous solution effect. A 2 × 2 × 1 Monkhorst–Pack *k*-point mesh was chosen to separate the Brillouin zone [43], and the valence electrons with a plane-wave basis was set to an energy cut-off of 400 eV. The convergence criteria for the geometry relaxation and electronic energy were set to 0.01 eV/Å and 10^−6^ eV, respectively. The Gaussian smearing method with a finite temperature width of 0.05 eV was implemented to improve the convergence of states near the Fermi level.

**Figure 1:**
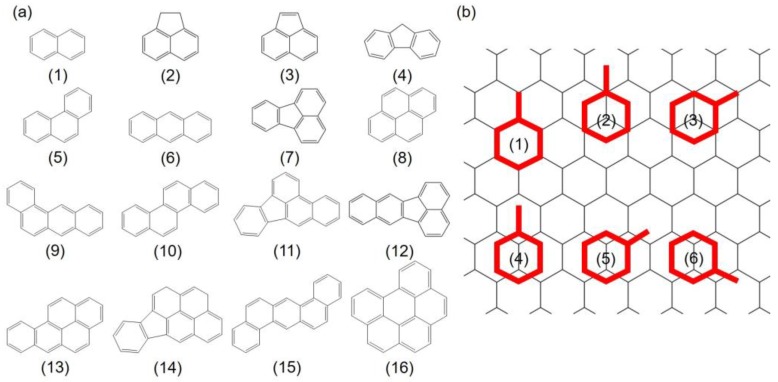
(**a**) Molecular structures of 16 PAHs selected in this study; (**b**) Six initial adsorption configurations of PAHs onto Gr depend on the symmetry of the PAHs: (1) refers to “hollow” site, (2) and (3) are “top” sites, and (4) to (6) indicate “bridge” sites, respectively. Gr is shown as a line model to increase the legibility. Red: PAHs molecules; black: Gr.

A total of 16 PAHs containing 2 to 6 fused rings with a wide range of molecular weights (128.2–278.4 g/mol) and C:H ratio (1.20–1.83) were selected in this study, as enlisted in Table 1 and Figure 1a.

The adsorption system which contains one PAH molecule was constructed to understand the behavior of PAHs adsorption. Previous study by Rajesh et al. [44] has shown that the aromatic rings favor a paralleled configuration when adsorbing onto Gr. Herein, the initial adsorption configurations were constructed by aligning the benzene rings in the PAHs on top of a parallel Gr to define the different adsorption sites. For each benzene ring, six sites were considered in the present study: one “hollow” site with the benzene ring of the PAHs located on top of and coincident with a hexagonal ring of Gr, two “top” sites with the center of the benzene ring on top of a carbon atom, and three “bridge” sites with the center of the benzene in the middle of C–C bond respectively, as shown in Figure 1b. The initial distances between PAHs and Gr were set to approximately 3.2 Å. To quantitatively describe the adsorption strength, the adsorption energy of above PAHs onto Gr is defined as Equation (1):

*E*_ad_ = *E*_PAH/Gr_ − *E*_PAH_ − *E*_Gr_, (1)

where *E*_PAH/Gr_, *E*_PAH_ and *E*_Gr_ stand for the total energy of the PAH adsorbed Gr, and energies of the isolated PAH and Gr sheet, respectively. A more negative *E*_ad_ indicates a more stable PAH/Gr system.

To examine the effect of strain on the adsorption behaviors of PAHs onto Gr, biaxial strains within the lateral plane varying from −2% to 10% with an interval of 1% were applied. The lattice constant of pristine Gr in strain-free condition is predicted to be *a*_0_ = 2.47 Å by DFT calculations, which agrees well with previous reported value [45]. Therefore, the lattice constant of strained Gr is set to *a* = (1 + *ε*) × *a*_0_.

### 2.2. Ab-Initio Molecular Dynamics (AIMD) Simulations

AIMD simulations of PAHs interacting with Gr were carried out using VASP [34,35,36] within the PBE-GGA method [37]. The total time of simulation was 9 ps with each time step setting to 1.0 fs. The plane wave energy cut-off was set to 400 eV. To capture the dynamic adsorption process of PAHs onto Gr, we set the Nosé–Hoover thermostat [46] controlling the temperature to 298 K within the canonical ensembles (NVT). For the consistency with DFT calculations, we also employed the DFT-D3 correction method to describe the van der Waals interactions in the adsorption systems [39,40,41].

## 3. Results

### 3.1. Adsorption Configurations

**Figure 2:**
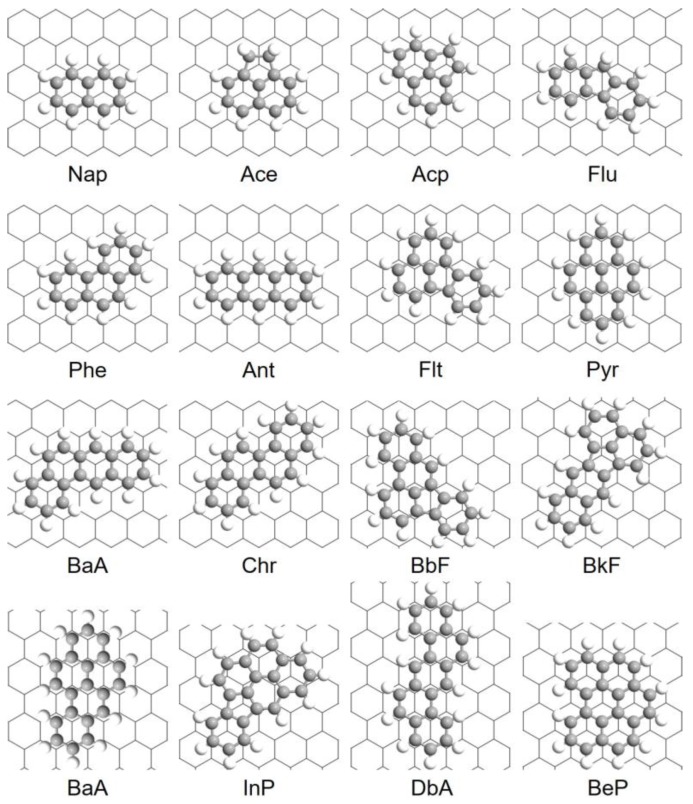
The most stable configurations of PAHs adsorbed onto Gr predicted by DFT calculations.

The most stable configurations of 16 PAHs adsorption onto Gr are summarized in Table 2 and Figure 2. As noted in Table 2, different PAHs may favor different adsorption (either “top” or “bridge”) sites on Gr, and there is no clear correlation between the site preference and adsorption energy. For instance, the “top” configuration is the most stable for Nap and Ace adsorption due to the maximized π–π interaction while “bridge” configuration may be preferred when it comes to larger PAHs (for example, BbF and BaP). The equilibrium distances between the PAH and Gr vary from 3.38 Å to 3.51 Å, and previous studies [47,48,49,50,51] have shown that the interaction between neutral aromatic molecules and Gr is determined by the joint interactions between Pauli repulsion, π–π interaction, short-range electrostatic Coulombic interactions, and van der Waals interactions at these equilibrium distances.

### 3.2. Adsorption Engertics

**Figure 3:**
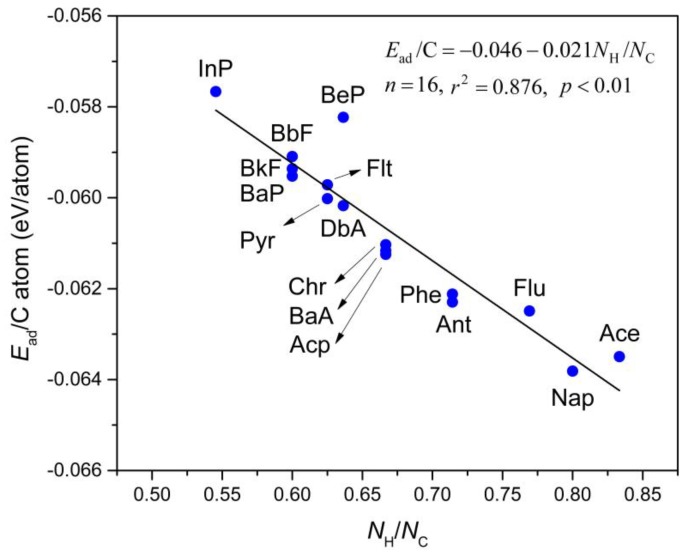
The relationship between adsorption energy per carbon atom (*E*_ad_/C atom; in eV/atom) and H:C ratio (*N*_H_/*N*_C_) of selected PAHs molecules. The straight line is fitted to Equation (2) which is shown in the inset.

Figure 3 depicts the computed *E*_ad_/C atom values, which is a linear relationship as a function of the H:C ratio in the PAHs molecules (*N*_H_/*N*_C_). This relationship directly fits to the equation shown as Equation (2):

*E*_ad_ = −0.046 − 0.021*N*_H_/*N*_C_, (2)

where *N*_H_ and *N*_C_ refer to the numbers of H and C atoms in the PAHs molecules, respectively. In this regard, when *N*_H_/*N*_C_ = 0 (i.e., the carbon atoms are the only component of the adsorbed molecule), the extrapolated *E*_ad_/C atom value will give the interlayer cohesive energy between two layers in graphite [57]. A value of 43 meV/atom was measured in a wetting experiment by Girifalco group [58]. More recently, Zacharia et al. [57] obtained the reference experimental value of approximately 52 ± 5 meV/atom for this property which was determined by thermal desorption analyses. The estimated value is approximately 46.0 meV/atom by PBE-D3 functional, with a small deviation of only ~3 meV/atom from the experimental result reported by Girifalco et al. [58] and ~6 meV/atom by Zacharia et al. [57], which verifies the reliability of the employed computational method. It is also important to note that this estimation is in great agreement with other values obtained from previous PBE-D3 and vdW-DF calculations (43–48 meV/atom) [59,60]. Therefore, we conclude that the selected methodology is reliable to examine the adsorption behavior of PAHs onto Gr.

The calculated adsorption energies between the PAHs and Gr using PBE-D3 and LDA are summarized in Table 2. Both the PBE-D3 and LDA calculation results demonstrate a similar trend: the adsorption energies become larger with the increasing total number of atoms in each of the PAHs. Among these adsorbates, Nap has the minimum adsorption energy with Gr (−0.638 eV by PBE-D3 and −0.353 eV by LDA), which implies the relatively weak interaction strength between Nap and Gr. It is also worth noting that the PBE-D3 results give larger adsorption energies as compared to LDA. Similar correlations between PBE-D3 and corresponding LDA energies with van der Waals corrections also have been witnessed in various biomolecular systems [61,62,63]. In addition, the energy differences between various adsorption configurations of the PAH/Gr systems are up to ~0.11 eV (see Table S1 in Supplementary). Therefore, the diffusion energy pathways of these aromatic molecules adsorbed onto Gr are very flat [31,64]. In this regard, PAHs are anticipated to diffuse and rotate (self-arranging) freely on the Gr under low and room temperature, but still adsorbed with large adsorption energies.

Furthermore, Equation (2) may be used as a direct method to predict the adsorption interactions between Gr and neutral and unsubstituted PAHs. Intriguingly, this equation also has been successfully applied to PAHs with five-membered ring or saturated moieties, such as Ace (C_12_H_10_), Acp (C_12_H_8_), Flu (C_13_H_10_) and Flt (C_16_H_10_). The predicted adsorption energies of these molecules are of −0.771 eV, −0.728 eV, −0.817 and −0.956 eV, respectively, which agree perfectly with the calculated values by PBE-D3 method (−0.762, −0.735, −0.812 and −0.955 eV, respectively).

### 3.3. Correlation between E_ad_ and logK_ow_

As suggested by various previous studies [65,66,67], hydrophobic interaction plays an essential part in PAH adsorption. The logarithm of the octanol/water partition coefficient (log*K*_ow_) is usually used to depict the hydrophobicity of analytes. The correlation between log*K*_ow_ and adsorption energies of the analytes onto Gr was studied to estimate the influence of hydrophobicity on the adsorption strength. log*K*_ow_ describes the ratio of analyte’s concentrations between two solutions: the octanol and water. The log*K*_ow_ values of the studied PAHs substantially rise with the increasing number of total atoms in the analytes according to a considerable amount of previous experimental results [52,53,54,55,56]. Thus, a larger value of log*K*_ow_ indicates a stronger hydrophobicity of the analytes. This correlation can be expressed by Equation (3):

*E*_ad_ = 0.03 − 0.19log*K*_ow_, *n* = 16, *r*^2^ = 0.978, *p* < 0.01. (3)

**Figure 4:**
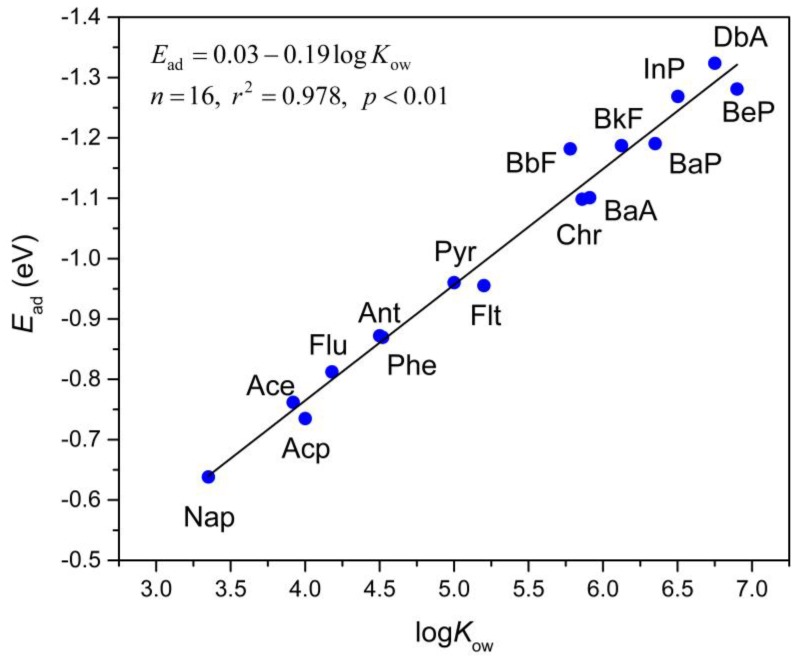
The correlation between the adsorption energy (*E*_ad_, in eV) and logarithm of the octanol/water partition coefficient (log*K*_ow_) of selected PAHs molecules. The straight line is fitted to Equation (3) which is shown in the inset.

The adsorption energies enhance with the increase of log*K*_ow_, indicating that the hydrophobic interactions between PAHs and Gr notably contribute to adsorption. As shown in Figure 4, the adsorption energy is in a linear correlation with the value of log*K*_ow_ for PAHs. This linear correlation suggests that the adsorption strength of PAHs onto Gr is primarily controlled by the hydrophobic interactions.

### 3.4. Electronic Properties

**Figure 5:**
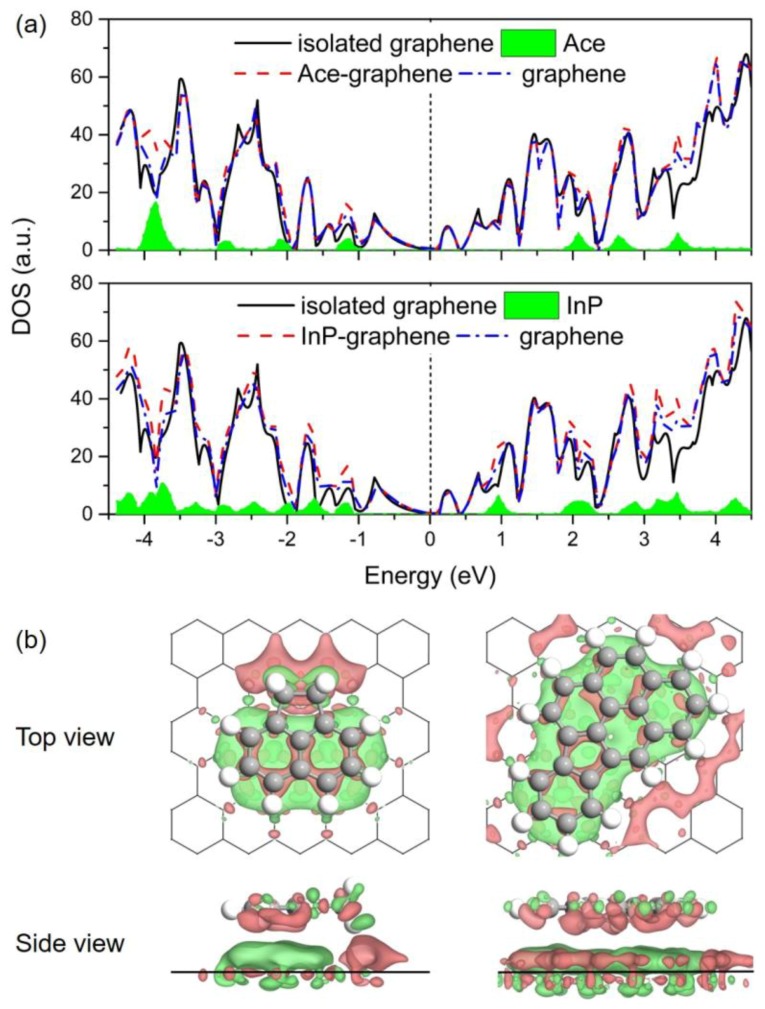
(**a**) Calculated density of states (DOS) for Ace/Gr (up) and InP/Gr (down) systems. The Fermi level was set as zero; (**b**) Differential electron density (Δ*ρ*) for Ace/Gr (left) and InP/Gr (right) adsorption systems. Isosurface contours of electron density differences are drawn at ±0.001 e/Bohr^3^. The electron accumulation and depletion are depicted in red and green colors, respectively.

The effects of adsorbed PAHs on the electronic properties of Gr are investigated in this section. Figure 5a depicts the comparison of density of states (DOS) for the Ace/Gr and InP/Gr adsorption systems prior to and post the adsorption. The total DOS near the Fermi level was almost a direct superposition by the partial DOSs of PAH molecule and Gr for both adsorption systems. No significant difference is observed for the DOS of Gr after the PAHs adsorption. Additionally, as shown in Table 2, Bader charge analysis [68] of these adsorption systems shows that there is little charge transfer from PAHs to Gr after the interaction, with the values ranging from 0.05 e to 0.08 e. Based on these results, it is reasonable to speculate that PAHs introduce electrons into Gr, behaving like mild n-dopants. It is also significant to note that the introduction of electrons and holes leads to enhanced conductive properties for the adsorbent. In addition, Figure 5b demonstrates the differential electron density (Δ*ρ*) prior to and post the adsorption, where the accumulation and depletion of electron density are displayed in red and green colors, respectively. From the Δ*ρ* isosurfaces, it is noticeable that the PAHs polarize the π-density in Gr through the intramolecular charge transfer exactly below the adsorption site; the adsorption sites result in electron-deficient states in this regard. This charge density redistribution accounts for the contribution of electrostatic interactions in the π–π stacking of aromatic molecules on Gr. Taking the above analysis together, the adsorption process of PAHs onto Gr is predicted to be physisorption.

### 3.5. Strain-Dependent PAHs Adsorption

**Figure 6:**
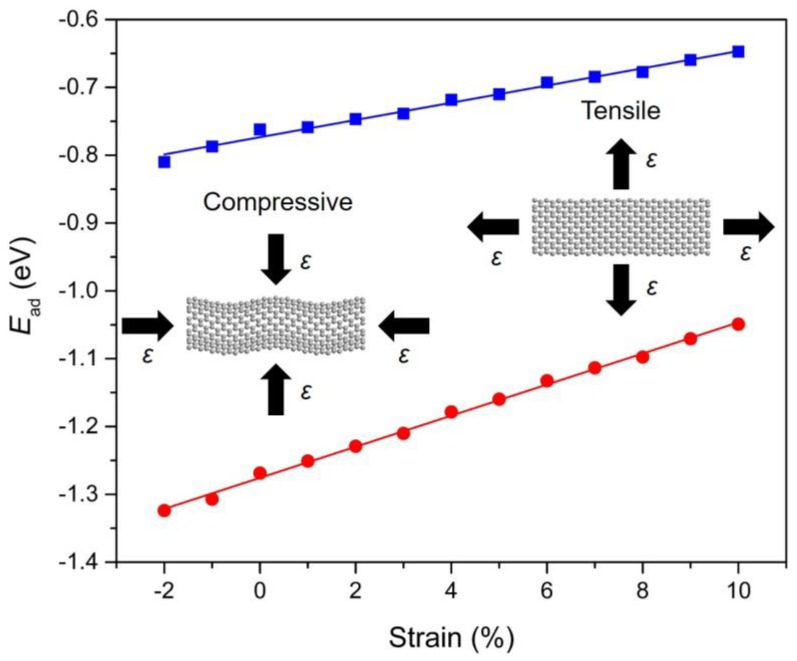
The correlation between the calculated adsorption energies (*E*_ad_; in eV) and the strain conditions (*ε*) for Ace (blue) and InP (red) adsorbed onto Gr. The blue and red straight lines are linearly fitted to the DFT calculated data points. The screenshots of Gr under compressive and tensile strains are illustrated as insets.

To investigate the role of strain engineering, we have also examined the adsorption behavior of PAHs onto Gr with a focus on the strain response of the adsorption. The calculation results indicate that the adsorption of PAHs onto Gr is highly sensitive to the strain condition that applied in the Gr layer (−2% to 10%). Figure 6 demonstrates the evolution of adsorption energy with respect to the applied strain for Ace/Gr and InP/Gr systems. The adsorption energy turns more negative with the compressive strain, indicating stronger adsorption strength when applied with the compressive strain. In terms of the compressive strain, it induces protuberance or ripples in the Gr. Therefore, the carbon atoms with a large curvature become more chemically reactive at specific locations, acting as preferred sites for PAHs adsorption. The adsorption strengths increase linearly with the increase of strains. It was also found that lattice expansion leads to weakened adsorption strength which can be interpreted by the fact that the tensile strain generally weakens the *sp*^2^ bonding between the carbon atoms in Gr and expands the lattice constants, thus decreases the interaction of π–π stacking between the PAHs and Gr. To our knowledge, there is no previous study demonstrating the manipulation of PAHs adsorptions on strain-free or strained Gr layers. It is expected that the predicted strain response of PAHs physical adsorption would trigger immediate interest and expand instant exploration for theoretical understanding and practical applications.

### 3.6. Dynamic Behavior of PAHs Adsorbed onto the Gr

**Figure 7:**
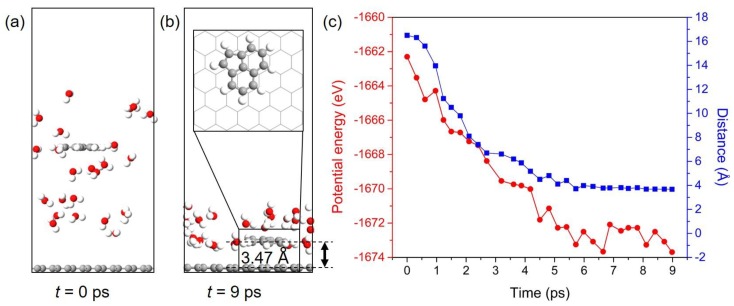
Configuration snapshots of the Ace/Gr adsorption system at (**a**) *t* = 0 ps and (**b**) *t* = 9 ps. (**c**) The potential energy (left) and distance (right) of Ace molecule adsorbed onto Gr with simulation time.

An exemplified model of Ace/Gr system was used to perform AIMD simulation to further study the dynamic behavior of PAHs adsorbed on Gr. One Ace molecule is placed in the water environment, and the distance between Ace and Gr is approximately 16 Å in the initial simulation box, as shown in Figure 7a. Ace molecule is finally adsorbed onto Gr and floated in a coplanar configuration after AIMD simulation of 9 ps. In addition, Ace molecule ends in an adsorption configuration that is different from the one obtained from DFT calculations. This can be explained by the temperature induced energy fluctuations in the AIMD simulation. This also indicates that the PAHs can diffuse or rotate on the Gr surface even under low or room temperature as the energy differences between various adsorption configurations are relatively small (see Supplementary Table S1 for details). According to simulation results, the equilibrium distance between Ace and Gr is approximately 3.47 Å, which is slightly larger than the value obtained from DFT calculation. The evolution of the potential energy and distance between Ace and Gr with respect to the time of the AIMD simulation is shown in Figure 7b. The potential energies and distances of the adsorption systems continuously reduce before the equilibrium state is achieved. PAHs have been proved to possess negligible or limited hydrogen bonding capacity [69]. Therefore, the adsorption of PAHs onto Gr cannot be directly promoted by the hydrogen-bonding interaction, whereas the water molecules around Ace can construct a hydrogen-bonding network. The adsorbates onto Gr can be further stabilized by the network through limiting their movement. Even though the hydrophobic interactions were demonstrated to be the primary driving force for PAHs adsorption, Ace can still interact with water molecules through the hydrogen bonds because of hydrogen atoms. Thereby, we conclude that the hydrogen bonds should exist between the interface of adsorbates and water, and they can further stabilize the adsorption systems. Therefore, the AIMD simulation indicates that the PAHs pollutants can be quickly captured by Gr and confirm the potential of Gr to adsorb and remove these aromatic pollutants.

## 4. Conclusions

In conclusion, DFT calculations and AIMD simulations have been employed to study the interaction and adsorption mechanism between Gr and PAHs. The adsorption energy was predicted to increase with the number of aromatic rings in the adsorbates and has a positive linear correlation with the hydrophobicity of PAHs. The adsorption process of PAHs onto Gr was estimated to be physical adsorption based on the analysis of DOS and differential electron density. We have also discovered that the PAHs’ adsorption is highly sensitive to the strained condition, that is, the applied compressive strain can tune and enhance the adsorption strength of these molecules onto Gr, which may be used as an ultrasensitive marker to detect these organic pollutants. In addition, the AIMD simulation indicates that Gr can act as sensitive adsorbent and achieve a fast adsorption process for PAHs, which confirms the remarkable performance of Gr for potential application in the capture and removal of PAHs.

## Figures and Tables

**Table 1:** Basic information of investigated polycyclic aromatic hydrocarbons (PAHs), including the name, abbreviation, CAS number and formula.

No.	Name	Abbreviation	CAS Number	Formula
1	Naphthalene	Nap	91-20-3	C_10_H_8_
2	Acenaphthene	Ace	83-32-9	C_12_H_10_
3	Acenaphthylene	Acp	208-96-8	C_12_H_8_
4	Fluorene	Flu	86-73-7	C_13_H_10_
5	Phenanthrene	Phe	85-01-8	C_14_H_10_
6	Anthracene	Ant	120-12-7	C_14_H_10_
7	Fluoranthene	Flt	206-44-0	C_16_H_10_
8	Pyrene	Pyr	129-00-0	C_16_H_10_
9	Benzo[a]anthracene	BaA	56-55-3	C_18_H_12_
10	Chrysene	Chr	218-01-9	C_18_H_12_
11	Benzo[b]fluoranthene	BbF	205-99-2	C_20_H_12_
12	Benzo[k]fluoranthene	BkF	207-08-9	C_20_H_12_
13	Benzo[a]pyrene	BaP	50-32-8	C_20_H_12_
14	Indeno[1,2,3-c,d]pyrene	InP	193-39-5	C_22_H_12_
15	Dibenz[a,h]anthracene	DbA	53-70-3	C_22_H_14_
16	Benzo[g,h,i]perylene	BeP	191-24-2	C_22_H_12_

**Table 2:** Density function theory (DFT) calculations predicted parameters of PAHs adsorption onto Gr, including the most stable configuration, adsorption energies by PBE-D3 and LDA (*E*_ad_; in eV), equilibrium distance (*d*_inter_; in Å), the logarithm of the octanol/water partition coefficient from the experiments (log*K*_ow_), as well as the charge transfer from Bader analysis (*Q*_PAH_; in e). Positive number of *Q*_PAH_ indicates that the charge is transferred from PAH to Gr.

No.	PAHs	Configurations	*E*_ad_ (eV)		*d*_inter_ (Å)	log*K*_ow_	*Q*_PAH_ (e)
			PBE-D3	LDA			
1	Nap	Top	−0.638	−0.353	3.45	3.37 [52]	0.051
2	Ace	Top	−0.762	−0.458	3.43	3.92 [52]	0.054
3	Acp	Top	−0.735	−0.427	3.44	4.00 [52]	0.052
4	Flu	Bridge	−0.812	−0.471	3.38	4.18 [52]	0.064
5	Phe	Top	−0.870	−0.491	3.44	4.57 [52]	0.061
6	Ant	Top	−0.872	−0.500	3.44	4.54 [52]	0.063
7	Flt	Bridge	−0.955	−0.537	3.45	5.22 [52]	0.064
8	Pyr	Bridge	−0.961	−0.540	3.50	4.88 [53,54]	0.062
9	BaA	Top	−1.101	−0.636	3.51	5.91 [52]	0.073
10	Chr	Top	−1.099	−0.628	3.45	5.86 [52]	0.072
11	BbF	Bridge	−1.182	−0.672	3.42	6.06 [54]	0.077
12	BkF	Top	−1.187	−0.673	3.42	6.12 [55]	0.077
13	BaP	Bridge	−1.190	−0.677	3.46	6.04 [52]	0.072
14	InP	Top	−1.269	−0.716	3.42	6.50 [52]	0.079
15	DbA	Bridge	−1.324	−0.757	3.43	6.75 [56]	0.080
16	BeP	Top	−1.281	−0.734	3.44	6.50 [52]	0.077

## Supplementary Materials

The following are available online at http://www.mdpi.com/1996-1944/11/5/726/s1, Table S1: Calculated adsorption energies of PAHs adsorption onto Gr for different initial configurations (refer to the initial configurations (1)–(6) illustrated in Figure 1) by PBE-D3 (*E*_ad_; in eV).
