# Supplementary material for: Polycyclic Aromatic Hydrocarbons Adsorption onto Graphene: A DFT and AIMD Study
Source: Materials (Basel). 2018 May 3;11(5):726. doi: 10.3390/ma11050726 (PMC5978103; doi:10.3390/ma11050726)
Supplement: Supplementary file 1 [file materials-11-00726-s001.pdf]

Supplementary Materials: Polycyclic Aromatic Hydrocarbons Adsorption onto Graphene: A DFT and AIMD Study

Bing Li, Pengfei Ou, Yulan Wei, Xu Zhang and Jun Song

Table S1. Calculated adsorption energies of polycyclic aromatic hydrocarbons (PAHs) adsorption onto Gr for different initial configurations (refer to the initial configurations (1)–(6) illustrated in Figure 1) by Perdew–Burke–Ernzerhof (PBE)-D3 (E_{ad}; in eV).

**PAHs**	E_{ad}					
	**Hollow (1)**	**Top (2)**	**Top (3)**	**Bridge (4)**	**Bridge (5)**	**Bridge (6)**
Nap	−0.598	**−0.638**	**−0.638**	−0.635	−0.632	−0.632
Ace	−0.714	**−0.762**	**−0.762**	−0.743	−0.757	−0.757
Acp	−0.696	−0.728	**−0.731**	−0.713	−0.730	−0.730
Flu	−0.779	−0.802	−0.801	−0.802	**−0.812**	−0.801
Phe	−0.813	−0.866	**−0.870**	−0.866	−0.862	−0.862
Ant	−0.819	**−0.872**	**−0.872**	−0.868	−0.867	−0.862
Flt	−0.916	−0.932	−0.954	−0.932	**−0.955**	**−0.955**
Pyr	−0.897	−0.950	−0.960	**−0.961**	−0.948	−0.950
BaA	−1.030	**−1.101**	−1.092	−1.093	−1.098	−1.092
Chr	−1.025	**−1.099**	−1.086	−1.092	−1.086	−1.091
BbF	−1.128	−1.158	−1.178	−1.158	−1.178	**−1.182**
BkF	−1.135	**−1.187**	−1.170	−1.166	−1.182	−1.174
BaP	−1.114	−1.189	−1.189	**−1.190**	−1.178	−1.177
InP	−1.222	**−1.269**	−1.262	−1.259	−1.254	−1.265
DbA	−1.236	−1.323	−1.323	−1.313	**−1.324**	−1.309
BeP	−1.190	**−1.281**	**−1.281**	−1.267	−1.274	−1.273
